# Community Health Worker Videoconferencing Interventions for Disease Management and Health Promotion: Protocol for a Scoping Review

**DOI:** 10.2196/55160

**Published:** 2024-11-07

**Authors:** Sonja France, Amy Dettori, Seda Ekici, Lilian McKinley, Sana Patel, Teresa Jewell, Mary E Crocker

**Affiliations:** 1 School of Medicine University of Washington Seattle, WA United States; 2 Division of Pulmonary & Sleep Medicine Seattle Children's Hospital Seattle, WA United States; 3 Department of Pediatrics School of Medicine University of Washington Seattle, WA United States; 4 Center for Respiratory Biology and Therapeutics Seattle Children's Research Institute Seattle, WA United States; 5 Department of Pediatrics Mount Nittany Medical Center State College, PA United States; 6 Department of Medicine Division of Allergy and Infectious Disease University of Washington Seattle, WA United States; 7 Health Sciences Library University of Washington Seattle, WA United States

**Keywords:** community health worker, lay health worker, telehealth, videoconferencing, intervention, digital divide, disease management, health promotion

## Abstract

**Background:**

Public health interventions delivered by community health workers (CHWs) have been proven effective in improving health outcomes across multiple fields, particularly in populations that are underserved by traditional health care systems. To date, little research is available about how CHW-led interventions could be successfully delivered virtually, despite many other health care services being offered via telehealth.

**Objective:**

This paper details a scoping review protocol that aims to assess the existing literature on CHW-led interventions using videoconferencing technology.

**Methods:**

The scoping review protocol was developed using the Joanna Briggs Institute’s guidelines on conduct of scoping reviews. Included papers will describe a direct intervention to manage disease or improve health, delivered by CHWs via videoconferencing. Multiple literature databases and gray literature will be searched. Abstracts and then full texts will be reviewed to determine inclusion by 2 independent reviewers; conflicts at each stage will be resolved by a third reviewer. A data extraction tool will be used by reviewers to independently chart data from included studies; results will be reviewed for accuracy by a second reviewer. Included papers will be analyzed to identify the breadth of the available evidence, including barriers, facilitators, effectiveness, and best practices described in the literature. Data will be summarized in a narrative review.

**Results:**

This study commenced in April 2022, and the study protocol was finalized in November 2022. A preliminary search of the published literature (excluding gray literature) in July 2024 revealed 276 reports after removal of duplicates. The formal literature search will commence in August 2024, with results available by December 2024. We intend to publish results in the academic literature as well as creating a report accessible to nonacademic and community audiences.

**Conclusions:**

This review will illuminate the breadth of the evidence available on CHW videoconferencing interventions, with specific focus on strategies for implementation success and equity of access. and will be of great value to organizations that offer CHW services.

**International Registered Report Identifier (IRRID):**

DERR1-10.2196/55160

## Introduction

Community health workers (CHWs) are workers who are not health care professionals but are trained to provide certain health interventions in their communities [1,2]. CHWs play pivotal roles in promoting health in a variety of settings and are especially recognized for their usefulness in resource-limited contexts. They are called upon to complete a broad variety of tasks, including providing education, connecting patients to resources, helping individuals overcome barriers to care, providing basic medical treatments, collecting health data, and providing psychosocial support [1,2]. Health interventions led by CHWs have been effective at improving outcomes in several areas including maternal and child health, infectious disease prevention, immunization, and chronic disease management [3]. They have also proved effective at reducing health disparities and improving the health of populations who face difficulties accessing care in traditional health care models [4-9].

CHW programs have long been using mobile health (mHealth) technology, which involves the use of mobile devices and apps to support communication between the CHW and the patient [10-12]. More recently, the growing interest in and demand for telehealth services, in part spurred by the COVID-19 pandemic, have raised the question of whether CHW services could also be adapted to use videoconferencing platforms [13,14]. The use of video presents an intriguing alternative to telephone-based CHW interventions, given that the ability of the patient to see the CHW face-to-face might enhance CHW trustworthiness and rapport [15], which are advantages in the essential CHW task of providing psychosocial support [2].

However, the use of video technology in disadvantaged populations creates a risk of widening the “digital divide,” referring to disparities in access to digital technology that are typically associated with socioeconomic factors [16-18]. For example, disadvantaged populations are more likely to encounter barriers such as lack of access to equipment or internet connectivity and low technological literacy, which are further exacerbated by language barriers [19-22]. The barriers to videoconferencing are likely greater than those in accessing other mHealth interventions, due to the increased internet bandwidth and device technology required, as well as the complexity of video apps. Conversely, if successful virtual CHW interventions can be developed, they could have great benefit, including potentially lower costs, reduced time required, and increased participant satisfaction, compared with in-person CHW services. If executed in a way that is sensitive to barriers faced by marginalized populations, they could also potentially increase access for participants who would not avail themselves of in-person CHW services [16,23]. These benefits would be relevant even in the post–COVID-19 era.

We sought to better understand the scope of current evidence regarding CHW videoconferencing interventions to determine how this evidence could be applied toward the development of high-quality CHW telehealth interventions that are sensitive to potential barriers faced by marginalized groups. To our knowledge there are no existing reviews addressing the delivery of videoconferencing interventions specifically by CHWs. We focus on videoconferencing due to the potential advantages of lower costs and time required and increased patient access and satisfaction, and given that several reviews of mHealth CHW interventions exist [10-12]. Therefore, the following research question of this scoping review was developed: What is known about videoconferencing community health worker interventions designed to improve health? Our population of interest is all persons regardless of age, gender, or background, and we will consider interventions studied in any context (including geographic location and language).

Our specific objectives are to determine (1) the kinds of health issues for which CHW videoconferencing interventions have been used, (2) barriers and facilitators to the CHW interventions, (3) any existing indicators of effectiveness of videoconferencing interventions, and (4) any identified best practices for CHW videoconferencing interventions. A scoping review was best suited to this research question because we sought to characterize the scope of evidence available rather than to statistically compare outcomes.

## Methods

We developed our protocol based on the guidelines laid out in the Joanna Briggs Institute’s guidelines on conduct of scoping reviews [24,25], and our reporting will follow the PRISMA-ScR (Preferred Reporting Items for Systematic Reviews and Meta-Analyses extension for Scoping Reviews) [26] and the checklist for reporting items for scoping review protocols developed by Joanna Briggs Institute (Multimedia Appendix 1) [25]. Important protocol updates will be reported in our final scoping review report. A preliminary search of relevant databases was conducted and no current or in-progress systematic or scoping reviews on the topic were identified.

### Eligibility Criteria

Papers will be reviewed if they meet each of the three inclusion criteria: (1) the paper describes an intervention designed to directly improve health, (2) the intervention is performed by a CHW, and (3) the intervention includes the use of videoconferencing (see Table 1 for further detail). We defined videoconferencing as a real-time interaction between the CHW and the participant with video and sound [27] (importantly excluding from this definition other forms of telehealth and mHealth, such as app-based, text message, or telephone-only interventions).

**Table 1:** Explanation of inclusion criteria for scoping review of community health worker videoconferencing interventions for disease management and health promotion.

Inclusion criteria	Explanation/important exclusions
Describes a program or intervention designed to improve health	This does not include training programs that do not involve delivering services.
Intervention is performed by a CHW^a^	We will not hold to any strict definition of CHW. We will include any abstract that self-defines as community health worker, lay health worker, promotor, etc.
Includes the use of videoconferencing	Videoconferencing is defined as a real-time interaction with video and sound, with the CHW on one end and participant on the other. This does not equal app-based, text-message based, or telephone-only activities that do not include a program or intervention that is designed to improve health and performed by a CHW.

^a^CHW: community health worker.

We adopted the definition of CHW outlined by Ballard et al [1] as “any lay health workers who:

1. Live in the area they serve
2. Are primarily based in the community (as opposed to a health facility)
3. Belong to the formal health system (i.e., are managed by the government or an implementing NGO)
4. Perform tasks related to health care delivery, and
5. Have received organized training but may not have received formal or paraprofessional certification or tertiary education degree” [1].

However, during our preliminary search we noted that the vast majority of papers did not provide enough information to verify whether this definition was met. Therefore, in order to create the most sensitive search strategy, we will include papers that self-define as involving CHWs or workers of other titles analogous to CHWs (Multimedia Appendix 2).

We will consider all sources of evidence for this review, including primary studies, literature reviews, opinions, letters, and guidelines. We will also include gray literature, as many community health interventions may be described on program or government sites and not captured in academic databases.

Exclusion criteria are failure to meet any of the inclusion criteria detailed in Table 1 or insufficient information in full-text review to determine whether each inclusion criterion is met (eg, if we are unable to determine whether there is video during the CHW-participant interactions). No studies will be excluded based on the date of publication, language of text, geographic location, outcome examined, or evidence type.

### Search Strategy

A research librarian at the University of Washington (author TJ) was consulted to guide the literature search strategy. Our search was structured around the use of 2 concepts: CHWs and videoconferencing. Based on prior knowledge of the literature and a preliminary literature search, we developed a list of keywords synonymous with each of these concepts. In addition, the list of synonyms for “community health worker” was augmented by consulting the MeSH (Medical Subject Headings)- and Emtree-controlled vocabulary and was expanded using proximity operators for databases in which this was an option. We then developed these lists into a full search strategy (Multimedia Appendix 2). An initial trial of the search string was run on PubMed and determined to successfully capture our target data. A weakness of the search string used was that it did capture papers describing remote training programs for CHWs, which are outside the scope of this review. However, it was determined that the volume of captured studies was low enough that these could be excluded in the abstract-screening stage. The search string was translated to the other databases (Multimedia Appendix 2). The databases PubMed, CINAHL, Embase, Global Health (EBSCO), Web of Science, Global Index Medicus, and the Cochrane Library will be searched for published literature. A gray literature search will be conducted using the following sources: IGO/search from the American Library Association Government Documents Caucus, a general Google search limited to .gov sites and .org sites, NIH RePORTER (which lists ongoing National Institutes of Health–funded projects), the Patient-Centered Outcomes Research Institute (which conducts studies on comparative clinical effectiveness research focusing on outcomes important to patients), Social Work Abstracts (EBSCO), Social Services Abstracts (ProQuest), and Sociological Abstracts (ProQuest). Reference lists of included papers will be reviewed, and additional papers may be added through snowball sampling during the data extraction phase.

### Data Management

After all searches have been performed, database search results will be uploaded to Mendeley (Elsevier Ltd) where duplicate papers will be removed. Citations will then be exported to RIS format and imported to Rayyan (Rayyan Sytems, Inc) [28] for screening. In the case of Google searches, results will be reviewed and duplicates removed by hand before uploading to Rayyan.

### Study Selection

All papers will undergo review to determine whether they meet inclusion criteria using the Rayyan software. For papers with an abstract, the abstract will first be screened. Papers whose abstracts meet inclusion criteria will progress to the full-text screening. We will also screen the full text of any papers whose abstracts do not include sufficient information to verify that all inclusion criteria are present. Each abstract will be screened by 2 different reviewers, blinded to the result of the other reviewer, and the paper will progress to the full-text screening when both reviewers agree. Conflicts will be resolved by a third-person tiebreaker. Papers that do not have an abstract will automatically be included in the full-text review stage. In preparation for abstract screening, a trial run will be conducted in which all reviewers screen the same 20 abstracts and then meet to discuss disagreements and clarify methods.

Full-text review will then be completed, again with 2 reviewers screening each paper in a blinded fashion using Rayyan. Conflicts will be resolved by a third, blinded reviewer. At the full-text review stage, if all inclusion criteria cannot be confirmed, the paper will be excluded. Reasons for exclusion of sources of evidence that do not meet the inclusion criteria at this stage will be recorded and reported in the scoping review.

The results of the search and the study selection process will be reported in full in the final scoping review and presented in a PRISMA-ScR flow diagram [26]. In addition, we will also include a list of citations for excluded studies and their reason for exclusion in the final review.

### Data Extraction

Data will be extracted from papers included in the scoping review using a data extraction tool developed by the reviewers, with 1 reviewer independently performing the data extraction and a second reviewer verifying the data. The draft data extraction tool (provided in Multimedia Appendix 3) will be pilot-tested on 2 papers by all reviewers prior to proceeding. The tool will be modified and revised as necessary during the process of extracting data. Modifications will be detailed in the scoping review. Any disagreements that arise between the reviewers will be resolved through discussion.

### Data Analysis

The data extraction tool will allow included papers to be distilled into the important points. Our analysis will include both descriptive statistics and a narrative review to summarize findings and draw conclusions. We will describe the number of papers, publication dates, country of origin or language, and what target populations or health issues are addressed in the papers in tabular format. We will then narratively describe our findings relating to the objectives of the review, including patterns in barriers and facilitators to the success of virtual programs, perceived drawbacks and benefits of virtual programs, and a summary of advice or best practices described in the literature. Depending on the number and breadth of papers included, we will consider separate subgroup analyses according to country income level (eg, high and upper-middle income vs low and lower-middle income countries) and type of concern targeted by the CHW intervention (ie, medical disease, mental health, social concerns, etc). As is typical of scoping reviews, we will not assess paper quality during data extraction as our goal is to cover the breadth of evidence available on the research question [24].

## Results

This study began in April 2022. The final study protocol was completed in November 2022. A preliminary search of the published literature (excluding gray literature) was performed in July 2024 and revealed 276 reports after removal of duplicates. The formal literature search is expected to commence in August 2024, with results projected to be available in December 2024. We intend to publish a manuscript in the public health literature to describe our conclusions to an academic audience, which should be ready for submission in 2025. We also intend to create a report that is accessible to nonacademic and community audiences detailing our findings.

## Discussion

The scoping review described in this protocol aims to elucidate the breadth of evidence available on videoconferencing interventions led by CHWs. We will focus our discussion of the results on effectiveness of studied interventions, how interventions should be delivered, what factors are important in their success or failure, and how to ensure access for underresourced individuals. Our planned subgroup analyses will help compare and contrast various approaches between programs with different target populations and health concerns.

We anticipate that our review will identify a relatively small number of peer-reviewed manuscripts describing the use of videoconferencing by CHWs, but that more examples may exist in the gray literature. Such a finding would reflect the need for additional high-quality studies examining such programs. In addition, we suspect that included studies may be primarily based in high-income countries, which would reflect the increased resources required to access videoconferencing interventions. Such a finding may spur future research on methods to reduce barriers to digital technologies for patients.

Of note, in this study we have focused on videoconferencing interventions delivered by CHWs, in order to explore the nature of these interventions and potentially uncover best practices for their delivery. Programs delivered using videoconferencing technology by nature require access to devices and web-based connectivity, which pose potential barriers for underresourced individuals. CHWs are often called upon to help address health disparities for such individuals [1,2], and videoconferencing interventions (especially if offered to the exclusion of other, more easily accessible modes of delivery) risk exacerbating existing health disparities, a concept known as the “digital divide” [13,29-31]. This review aims to spotlight practices used by CHW videoconferencing interventions to mitigate widening of this divide.

To our knowledge, this will be the first review to focus on the use of videoconferencing during CHW interactions with patients. However, existing literature exploring CHW use of mHealth interventions offers insights that may be applicable to CHW videoconferencing interventions. Specifically, mHealth interventions using apps and mobile devices can improve engagement of both the CHW and the client [10]. However, several challenges in adoption and feasibility of mHealth interventions have been identified, including difficulties with CHWs adopting the technologies (although this could be mitigated by adequate training), technical difficulties with hardware and software, and lack of culturally appropriate design [10,11]. Such challenges could present themselves to an even greater degree in CHW use of videoconferencing, given the fact that videoconferencing technology is potentially more difficult to use and resource-intensive than mHealth applications; our review will help characterize these differences.

Existing literature investigating the use of videoconferencing by health care providers other than CHWs can also be illuminating. For example, a systematic review of telemedicine programs (including synchronous telephone and videoconferencing) implemented within formal health care systems in US rural or low-income populations concluded that telemedicine is acceptable and feasible to these underresourced groups [32]. Advantages of telemedicine over in-person services included increased efficiency, reduced costs (in some cases), improved access for distant patient populations, and reduced missed appointments. Several potential barriers to implementation were identified, including reduced participant satisfaction with or interest in telehealth in some subpopulations, lack of technical expertise or quality broadband access, low patient digital literacy, and difficulty with establishing rapport remotely [32]. The use of teleconferencing by CHWs in similar populations may present similar advantages and challenges, and our review will add to the literature a greater understanding of these as it relates to the unique roles and capabilities of CHWs.

Our study design has a number of limitations that we have worked to mitigate. One such limitation is that the terms used to describe the concept of a CHW in the health literature are variable, and distinct terms are used to describe individuals with similar roles in different contexts. We have attempted to create a comprehensive, culturally inclusive search strategy to address these limitations. On the other hand, many reports do not explicitly describe the background or functions of individuals identified as CHWs, making it difficult to verify that the terms are used similarly across studies, which may complicate the interpretation of our results. Defining a comprehensive search to target the concept of videoconferencing is similarly challenging, as terms related to telehealth are inconsistently used and have considerable overlap. For example, videoconferencing services could be alternatively described using synonyms such as “virtual,” “remote,” or “teleconferencing,” but these terms are imprecise and also reference a variety of other types of telehealth services. Finally, we will not assess the quality of studies included in our analysis. We feel that this methodologic choice is both consistent with the format of a scoping review and pragmatic due to the inclusion of all study designs as well as gray literature.

In conclusion, this scoping review will characterize the evidence available regarding videoconferencing interventions delivered by CHWs to improve health, with a focus on best practices for implementation success as well as strategies to ensure equitable access. As the digital divide persists between those who have access to digital technology and those who do not, it is imperative that interventions using developing technologies ensure access to services for those who are the most vulnerable. CHWs have multiple strengths in reducing health disparities and promoting health among underresourced populations, and while videoconferencing presents an opportunity to expand their reach, it must not be at the sacrifice of those strengths. This review will provide evidence that will be invaluable to governmental and community-based organizations that offer CHW services, as well as researchers studying the effectiveness of these interventions. Ultimately, our results will enable the development of recommendations to improve likelihood of intervention success, while simultaneously avoiding widening the digital divide.

## Appendix

Multimedia Appendix 1 Joanna Briggs Institute scoping review protocol reporting checklist.

Multimedia Appendix 2 Search strings.

Multimedia Appendix 3 Data extraction table.

## Abbreviations

CHW: community health worker
MeSH: Medical Subject Headings
mHealth: mobile health
PRISMA-ScR: Preferred Reporting Items for Systematic Reviews and Meta-Analyses extension for Scoping Reviews

## Data Availability

All data generated or analyzed during this study will be included in the published paper for the final Scoping Review and its supplementary information files.
