# Supplementary material for: Community Health Worker Videoconferencing Interventions for Disease Management and Health Promotion: Protocol for a Scoping Review
Source: JMIR Res Protoc. 2024 Nov 7;13:e55160. doi: 10.2196/55160 (PMC11582480; doi:10.2196/55160)
Supplement: Multimedia Appendix 2 [file resprot_v13i1e55160_app2.pdf]

Search Strings and Terms

This is a Multimedia Appendix to a full manuscript published in the J Med Internet Res. titled “Community health worker videoconferencing interventions for disease management and health promotion: A protocol for a scoping review”

Embase; Elsevier

	Concept: Community Health	Concept: Videoconferencing
Subject Headings (Emtree)	'health auxiliary'/exp	'videoconferencing'/exp<br>'web conferencing'/exp<br>'video consultation'/exp
Free text terms	CHW<br>CHWs<br>(community NEXT/2 (health OR "health care" OR healthcare) NEXT/2 (worker* OR advisor* OR agent* OR aid* OR officer* OR practitioner* OR representative*))<br>((lay OR nonphysician OR "non-physician" OR auxiliary OR village) NEXT/2 (health OR "health care" OR healthcare) NEXT/2 worker*)<br>((health OR 'health care' OR healthcare) NEXT/2 (promoter* OR advocate*))<br>promotore*<br>promotora*<br>'outreach educator*'<br>(peer NEXT/2 (health OR "health care" OR healthcare) NEXT/2 educator*)<br>NPHW<br>((health OR 'health care' OR healthcare OR medical OR 'medical care') NEXT/2 auxiliar*)<br>(('social health' OR 'social healthcare' OR 'social health care') NEXT/2 activist*)	videoconferenc*<br>"video conferenc*"<br>"video visit*"<br>"virtual visit*"<br>"virtual intervention*"<br>"remote consult*"<br>"video consult*"<br>"videoconsult*"<br>"TIME intervention*"<br>(('telehealth'/exp OR telemedicine OR telehealth OR ehealth OR "e-health" OR "remote care") AND (video* OR virtual OR Zoom OR Skype OR FaceTime))

('health auxiliary'/exp OR CHW OR CHWs OR (community NEXT/2 (health OR 'health care' OR healthcare) NEXT/2 (worker* OR advisor* OR agent* OR aid* OR officer* OR practitioner* OR representative*)) OR ((lay OR nonphysician OR 'non-physician' OR auxiliary OR village) NEXT/2 (health OR 'health care' OR healthcare) NEXT/2 worker*) OR ((health OR 'health care' OR healthcare) NEXT/2 (promoter* OR advocate*)) OR promotore* OR promotora* OR 'outreach educator*' OR (peer NEXT/2 (health OR 'health care' OR healthcare) NEXT/2 educator*) OR NPHW OR ((health OR 'health care' OR healthcare OR medical OR 'medical care') NEXT/2 auxiliar*) OR (('social health' OR 'social healthcare' OR 'social health care') NEXT/2 activist*)**)**

AND

('videoconferencing'/exp OR 'web conferencing'/exp OR 'video consultation'/exp OR videoconferenc* OR "video conferenc*" OR "video visit*" OR "virtual visit*" OR "virtual intervention*" OR "remote consult*" OR "videoconsult*" OR "video consult*" OR "TIME intervention*" OR (('telehealth'/exp OR telemedicine OR telehealth OR ehealth OR "e-health" OR "remote care") AND (video* OR virtual OR Zoom OR Skype OR FaceTime))**)**

<u>PubMed</u>

	**Concept: Community Health**	**Concept: Videoconferencing**
Subject Headings (MeSH)	"community health workers"[Mesh]	Videoconferencing[Mesh]
Free text terms	"community health worker*"<br>"community health care worker*"<br>"community healthcare worker*"<br>CHW<br>CHWs<br>"community health advisor*"<br>"community health care advisor*"<br>"community healthcare advisor*"<br>"community health agent*"<br>"community health care agent*"<br>"community healthcare agent*"<br>"community health aid*"<br>"community health care aid*"<br>"community healthcare aid*"<br>"community health officer*"<br>"community health care officer*"<br>"community healthcare officer*"<br>"community health practitioner*"<br>"community health care practitioner*"<br>"community healthcare practitioner*"<br>"community health representative*"<br>"community health care representative*"<br>"community healthcare representative*"<br>"lay health worker*"<br>"lay health care worker*"<br>"lay healthcare worker*"<br>"health promoter*"<br>"health care promoter*"<br>"healthcare promoter*"<br>promotore*<br>promotora*<br>"health advocate*"<br>"health care advocate*"<br>"healthcare advocate*"<br>"outreach educator*"<br>"peer health educator*"<br>"peer health care educator*"<br>"peer healthcare educator*"<br>"nonphysician health worker*"<br>"nonphysician health care worker*"	videoconferenc*<br>"video conferenc*"<br>"video visit*"<br>"virtual visit*"<br>"virtual intervention*"<br>"remote consult*"<br>"video consult*"<br>videoconsult*<br>"TIME intervention*"<br>((Telemedicine[Mesh] OR telemedicine OR telehealth OR ehealth OR "e-health" OR "remote care") AND (video* OR virtual OR Zoom OR Skype OR FaceTime))

	"nonphysician healthcare worker*"<br>"non-physician health worker*"<br>"non-physician health care worker*"<br>"non-physician healthcare worker*"<br>NPHW*<br>"health auxiliar*"<br>"health care auxiliar*"<br>"healthcare auxiliar*"<br>"medical auxiliar*"<br>"medical care auxiliar*"<br>"auxiliary health worker*"<br>"auxiliary health care worker*"<br>"auxiliary healthcare worker*"<br>"social health activist*"<br>"social health care activist*"<br>"social healthcare activist*"<br>"village health worker*"<br>"village health care worker*"<br>"village healthcare worker*"	

("community health workers"[Mesh] OR "community health worker*" OR "community health care worker*" OR "community healthcare worker*" OR CHW OR CHWs OR "community health advisor*" OR "community health care advisor*" OR "community healthcare advisor*" OR "community health agent*" OR "community health care agent*" OR "community healthcare agent*" OR "community health aid*" OR "community health care aid*" OR "community healthcare aid*" OR "community health officer*" OR "community health care officer*" OR "community healthcare officer*" OR "community health practitioner*" OR "community health care practitioner*" OR "community healthcare practitioner*" OR "community health representative*" OR "community health care representative*" OR "community healthcare representative*" OR "lay health worker*" OR "lay health care worker*" OR "lay healthcare worker*" OR "health promoter*" OR "health care promoter*" OR "healthcare promoter*" OR promotore* OR promotora* OR "health advocate*" OR "health care advocate*" OR "healthcare advocate*" OR "outreach educator*" OR "peer health educator*" OR "peer health care educator*" OR "peer healthcare educator*" OR "nonphysician health worker*" OR "nonphysician health care worker*" OR "nonphysician healthcare worker*" OR "non-physician health worker*" OR "non-physician health care worker*" OR "non-physician healthcare worker*" OR NPHW* OR "health auxiliar*" OR "health care auxiliar*" OR "healthcare auxiliar*" OR "medical auxiliar*" OR "medical care auxiliar*" OR "auxiliary health worker*" OR "auxiliary health care worker*" OR "auxiliary healthcare worker*" OR "social health activist*" OR "social health care activist*" OR "social healthcare activist*" OR "village health worker*" OR "village health care worker*" OR "village healthcare worker*"**)**

AND

(Videoconferencing[Mesh] OR videoconferenc* OR "video conferenc*" OR "video visit*" OR "virtual visit*" OR "virtual intervention*" OR "remote consult*" OR "video consult*" OR "videoconsult*" OR "TIME intervention*" OR ((Telemedicine[Mesh] OR telemedicine OR telehealth OR ehealth OR "e-health" OR "remote care") AND (video* OR virtual OR Zoom OR Skype OR FaceTime)) **)**

Limits:

Note: for consistency's sake this includes quoted phrases that are not found in PubMed. PubMed handles those by searching them with AND between the terms. If those phrases are removed, there are 68 results.

("community health workers"[Mesh] OR "community health worker*" OR "community health care worker*" OR "community healthcare worker*" OR CHW OR CHWs OR "community health advisor*" OR "community health agent*"

OR "community healthcare agent*" OR "community health aid*" OR "community health officer*" OR "community health practitioner*" OR "community healthcare practitioner*" OR "community health representative*" OR "lay health worker*" OR "lay health care worker*" OR "lay healthcare worker*" OR "health promoter*" OR promotore* OR promotora* OR "health advocate*" OR "health care advocate*" OR "healthcare advocate*" OR "outreach educator*" OR "peer health educator*" OR "nonphysician health worker*" OR "nonphysician health care worker*" OR "nonphysician healthcare worker*" OR "non-physician health worker*" OR "non-physician health care worker*" OR "non-physician healthcare worker*" OR NPHW* OR "health auxiliar*" OR "health care auxiliar*" OR "healthcare auxiliar*" OR "medical auxiliar*" OR "auxiliary health worker*" OR "auxiliary health care worker*" OR "auxiliary healthcare worker*" OR "social health activist*" OR "village health worker*" OR "village health care worker*" OR "village healthcare worker*"**)**

AND

(Videoconferencing[Mesh] OR videoconferenc* OR "video conferenc*" OR "video visit*" OR "virtual visit*" OR "virtual intervention*" OR "remote consult*" OR "video consult*" OR "videoconsult*" OR "TIME intervention*" OR ((Telemedicine[Mesh] OR telemedicine OR telehealth OR ehealth OR "e-health" OR "remote care") AND (video* OR virtual OR Zoom OR Skype OR FaceTime)) **)**

<u>Cumulative Index to Nursing and Allied Health Literature (CINAHL); EBSCO</u>

	**Concept: Community Health**	**Concept: Videoconferencing**
Subject Headings (MH)	"Community Health Workers"	"Videoconferencing"<br>"Remote Consultation"
Free text terms	CHW<br>CHWs<br>((community W1 (health OR "health care" OR healthcare)) W1 (worker* OR advisor* OR agent* OR aid* OR officer* OR practitioner* OR representative*))<br>(((lay OR nonphysician OR "non-physician" OR auxiliary OR village) W1 (health OR "health care" OR healthcare)) W1 worker*)<br>((health OR 'health care' OR healthcare) W1 (promoter* OR advocate*))<br>promotore*<br>promotora*<br>((outreach OR (peer W1 (health OR "health care" OR healthcare))) W1 educator*)<br>NPHW<br>((health OR 'health care' OR healthcare OR medical OR 'medical care') W1 auxiliar*)<br>(('social health' OR 'social healthcare' OR 'social health care') W1 activist*)	videoconferenc*<br>"video conferenc*"<br>"video visit*"<br>"virtual visit*"<br>"virtual intervention*"<br>"remote consult*"<br>"video consult*"<br>"videoconsult*"<br>"TIME intervention*"<br>(((MH Telehealth) OR telemedicine OR telehealth OR ehealth OR "e-health" OR "remote care") AND (video* OR virtual OR Zoom OR Skype OR FaceTime))

((MH "Community Health Workers") OR CHW OR CHWs OR ((community W1 (health OR "health care" OR healthcare)) W1 (worker* OR advisor* OR agent* OR aid* OR officer* OR practitioner* OR representative*)) OR (((lay OR nonphysician OR "non-physician" OR auxiliary OR village) W1 (health OR "health care" OR healthcare)) W1 worker*) OR ((health OR 'health care' OR healthcare) W1 (promoter* OR advocate*)) OR promotore* OR promotora* OR ((outreach OR (peer W1 (health OR "health care" OR healthcare))) W1 educator*) OR NPHW OR ((health OR 'health care' OR healthcare OR medical OR 'medical care') W1 auxiliar*) OR (('social health' OR 'social healthcare' OR 'social health care') W1 activist*)**)**

AND

(MH ("Videoconferencing" OR "Remote Consultation") OR videoconferenc* OR "video conferenc*" OR "video visit*" **OR** "virtual visit*" OR "virtual intervention*" OR "remote consult*" OR "video consult*" **OR** "videoconsult*" OR "TIME intervention*" OR (((MH Telehealth) OR telemedicine OR telehealth OR ehealth OR "e-health" OR "remote care") AND (video* OR virtual OR Zoom OR Skype OR FaceTime)) **)**

Limits: on the Advanced Search screen, make sure that "Apply related words", "Also search within the full text of the articles", and "Apply equivalent subjects" are all un-checked.

PsycInfo; EBSCO

	**Concept: Community Health**	**Concept: Videoconferencing**
PsycInfo Subject Headings (DE)	-	"Videoconferencing"
MeSH terms (MA)	"Community Health Workers"	"Videoconferencing"
Free text terms	CHW<br>CHWs<br>((community W1 (health OR "health care" OR healthcare)) W1 (worker* OR advisor* OR agent* OR aid* OR officer* OR practitioner* OR representative*))<br>(((lay OR nonphysician OR "non-physician" OR auxiliary OR village) W1 (health OR "health care" OR healthcare)) W1 worker*)<br>((health OR 'health care' OR healthcare) W1 (promoter* OR advocate*))<br>promotore*<br>promotora*<br>((outreach OR (peer W1 (health OR "health care" OR healthcare))) W1 educator*)<br>NPHW<br>((health OR 'health care' OR healthcare OR medical OR 'medical care') W1 auxiliar*)<br>(('social health' OR 'social healthcare' OR 'social health care') W1 activist*)	videoconferenc*<br>"video conferenc*"<br>"video visit*"<br>"virtual visit*"<br>"virtual intervention*"<br>"remote consult*"<br>"video consult*"<br>"videoconsult*"<br>"TIME intervention*"<br>(((DE ("Telemedicine" OR "Online Therapy" OR "Teleconsultation" OR "Telepsychiatry" OR "Telepsychology" OR "Telerehabilitation")) OR (MA Telemedicine) OR telemedicine OR telehealth OR ehealth OR "e-health" OR "remote care") AND (video* OR virtual OR Zoom OR Skype OR FaceTime))

((MA "Community Health Workers") OR CHW OR CHWs OR ((community W1 (health OR "health care" OR healthcare)) W1 (worker* OR advisor* OR agent* OR aid* OR officer* OR practitioner* OR representative*)) OR (((lay OR nonphysician OR "non-physician" OR auxiliary OR village) W1 (health OR "health care" OR healthcare)) W1 worker*) OR ((health OR 'health care' OR healthcare) W1 (promoter* OR advocate*)) OR promotore* OR promotora* OR ((outreach OR (peer W1 (health OR "health care" OR healthcare))) W1 educator*) OR NPHW OR ((health OR 'health care' OR healthcare OR medical OR 'medical care') W1 auxiliar*) OR (('social health' OR 'social healthcare' OR 'social health care') W1 activist*)**)**

AND

((DE "Videoconferencing") OR (MA "Videoconferencing") OR videoconferenc* OR "video conferenc*" OR "video visit*" **OR** "virtual visit*" OR "virtual intervention*" OR "remote consult*" OR "video consult*" **OR** "videoconsult*" OR "TIME intervention*" OR (((DE ("Telemedicine" OR "Online Therapy" OR "Teleconsultation" OR "Telepsychiatry" OR "Telepsychology" OR "Telerehabilitation")) OR (MA Telemedicine) OR telemedicine OR telehealth OR ehealth OR "e-health" OR "remote care") AND (video* OR virtual OR Zoom OR Skype OR FaceTime)) **)**

Limits: on the Advanced Search screen, make sure that "Apply related words", "Also search within the full text of the articles", and "Apply equivalent subjects" are all un-checked.

Web of Science Core Collection

	Concept: Community Health	Concept: Videoconferencing
Topic<br>(searches title, abstract, author keywords, Keywords Plus®)	CHW<br>CHWs<br>((community NEAR/1 (health OR "health care" OR healthcare)) NEAR/1 (worker* OR advisor* OR agent* OR aid* OR officer* OR practitioner* OR representative*))<br>(((lay OR nonphysician OR "non-physician" OR auxiliary OR village) NEAR/1 (health OR "health care" OR healthcare)) NEAR/1 worker*)<br>((health OR "health care" OR healthcare) NEAR/1 (promoter* OR advocate*))<br>promotore*<br>promotora*<br>((outreach OR (peer NEAR/1 (health OR "health care" OR healthcare))) NEAR/1 educator*)<br>NPHW<br>((health OR "health care" OR healthcare OR medical OR "medical care") NEAR/1 auxiliar*)<br>(("social health" OR "social healthcare" OR "social health care") NEAR/1 activist*)	videoconferenc*<br>"video conferenc*"<br>"video visit*"<br>"virtual visit*"<br>"virtual intervention*"<br>"remote consult*"<br>"video consult*"<br>"videoconsult*"<br>"TIME intervention*"<br>(('telehealth'/exp OR telemedicine OR telehealth OR ehealth OR "e-health" OR "remote care") AND (video* OR virtual OR Zoom OR Skype OR FaceTime))

(CHW OR CHWs OR ((community NEAR/1 (health OR "health care" OR healthcare)) NEAR/1 (worker* OR advisor* OR agent* OR aid* OR officer* OR practitioner* OR representative*)) OR (((lay OR nonphysician OR "non-physician" OR auxiliary OR village) NEAR/1 (health OR "health care" OR healthcare)) NEAR/1 worker*) OR ((health OR "health care" OR healthcare) NEAR/1 (promoter* OR advocate*)) OR promotore* OR promotora* OR ((outreach OR (peer NEAR/1 (health OR "health care" OR healthcare))) NEAR/1 educator*) OR NPHW OR ((health OR "health care" OR healthcare OR medical OR "medical care") NEAR/1 auxiliar*) OR (("social health" OR "social healthcare" OR "social health care") NEAR/1 activist*)**)**

AND

(videoconferenc* OR "video conferenc*" OR **"video visit*" OR** "virtual visit*" OR "virtual intervention*" OR **"remote consult*" OR "video consult*" OR "videoconsult*" OR** "TIME intervention*" OR ((telemedicine OR telehealth OR ehealth OR "e-health" OR "remote care") AND (video* OR virtual OR Zoom OR Skype OR FaceTime)) **)**

Global Index Medicus

	**Concept: Community Health**	**Concept: Videoconferencing**
Subject Descriptors	mh:M01.526.485.067.080*	mh:SP2.021.167.010.120*
Title, abstract, subject<br>(tw:())	"community health worker"<br>"community health workers"<br>"community health care worker"<br>"community health care workers"<br>"community healthcare worker"<br>"community healthcare workers"<br>CHW<br>CHWs<br>"community health advisor"<br>"community health advisors"<br>"community health care advisor"<br>"community health care advisors"<br>"community healthcare advisor"<br>"community healthcare advisors"<br>"community health agent"<br>"community health agents"<br>"community health care agent"<br>"community health care agents"<br>"community healthcare agent"<br>"community healthcare agents"<br>"community health aid"<br>"community health aids"<br>"community health care aid"<br>"community health care aids"<br>"community healthcare aid"<br>"community healthcare aids"<br>"community health officer"<br>"community health officers"<br>"community health care officer"<br>"community health care officers"<br>"community healthcare officer"<br>"community healthcare officers"<br>"community health practitioner"<br>"community health practitioners"<br>"community health care practitioner"<br>"community health care practitioners"<br>"community healthcare practitioner"<br>"community healthcare practitioners"<br>"community health representative"<br>"community health representatives"<br>"community health care representative"<br>"community health care representatives"<br>"community healthcare representative"<br>"community healthcare representatives"<br>"lay health worker"<br>"lay health workers"<br>"lay health care worker"<br>"lay health care workers"	videoconferenc*<br>"video conference"<br>"video conferences"<br>"video conferencing"<br>"video visit"<br>"video visits"<br>"video visitation"<br>"video visitations"<br>"virtual visit"<br>"virtual visits"<br>"virtual visitation"<br>"virtual visitations"<br>"virtual intervention"<br>"virtual interventions"<br>"remote consult"<br>"remote consults"<br>"remote consultation"<br>"remote consultations"<br>"remote consulting"<br>"video consult"<br>"video consults"<br>"video consultation"<br>"video consultations"<br>"video consulting"<br>"videoconsult"<br>"videoconsults"<br>"videoconsultation"<br>"videoconsultations"<br>"videoconsulting"<br>"TIME intervention"<br>"TIME interventions"<br>(((mh: H02.403.840*) OR telemedicine OR telehealth OR ehealth OR "e-health" OR "remote care") AND (video* OR virtual OR Zoom OR Skype OR FaceTime)))

	"lay healthcare worker"<br>"lay healthcare workers"<br>"health promoter"<br>"health promoters"<br>"health care promoter"<br>"health care promoters"<br>"healthcare promoter"<br>"healthcare promoters"<br>promotore<br>promotores<br>promotora<br>promotoras<br>"health advocate"<br>"health advocates"<br>"health care advocate"<br>"health care advocates"<br>"healthcare advocate"<br>"healthcare advocates"<br>"outreach educator"<br>"outreach educators"<br>"peer health educator"<br>"peer health educators"<br>"peer health care educator"<br>"peer health care educators"<br>"peer healthcare educator"<br>"peer healthcare educators"<br>"nonphysician health worker"<br>"nonphysician health workers"<br>"nonphysician health care worker"<br>"nonphysician health care workers"<br>"nonphysician healthcare worker"<br>"nonphysician healthcare workers"<br>"non-physician health worker"<br>"non-physician health workers"<br>"non-physician health care worker"<br>"non-physician health care workers"<br>"non-physician healthcare worker"<br>"non-physician healthcare workers"<br>NPHW<br>NPHWs<br>"health auxiliary"<br>"health auxiliaries"<br>"health care auxiliary"<br>"health care auxiliaries"<br>"healthcare auxiliary"<br>"healthcare auxiliaries"<br>"medical auxiliary"<br>"medical auxiliaries"<br>"medical care auxiliary"<br>"medical care auxiliaries"<br>"auxiliary health worker"<br>"auxiliary health workers"<br>"auxiliary health care worker"	

	"auxiliary health care workers"<br>"auxiliary healthcare worker"<br>"auxiliary healthcare workers"<br>"social health activist"<br>"social health activists"<br>"social health care activist"<br>"social health care activists"<br>"social healthcare activist"<br>"social healthcare activists"<br>"village health worker"<br>"village health workers"<br>"village health care worker"<br>"village health care workers"<br>"village healthcare worker"<br>"village healthcare workers"	

((mh:M01.526.485.067.080*) OR "community health worker" OR "community health workers" OR "community health care worker" OR "community health care workers" OR "community healthcare worker" OR "community healthcare workers" OR CHW OR CHWs OR "community health advisor" OR "community health advisors" OR "community health care advisor" OR "community health care advisors" OR "community healthcare advisor" OR "community healthcare advisors" OR "community health agent" OR "community health agents" OR "community health care agent" OR "community health care agents" OR "community healthcare agent" OR "community healthcare agents" OR "community health aid" OR "community health aids" OR "community health care aid" OR "community health care aids" OR "community healthcare aid" OR "community healthcare aids" OR "community health officer" OR "community health officers" OR "community health care officer" OR "community health care officers" OR "community healthcare officer" OR "community healthcare officers" OR "community health practitioner" OR "community health practitioners" OR "community health care practitioner" OR "community health care practitioners" OR "community healthcare practitioner" OR "community healthcare practitioners" OR "community health representative" OR "community health representatives" OR "community health care representative" OR "community health care representatives" OR "community healthcare representative" OR "community healthcare representatives" OR "lay health worker" OR "lay health workers" OR "lay health care worker" OR "lay health care workers" OR "lay healthcare worker" OR "lay healthcare workers" OR "health promoter" OR "health promoters" OR "health care promoter" OR "health care promoters" OR "healthcare promoter" OR "healthcare promoters" OR promotore OR promotores OR promotora OR promotoras OR "health advocate" OR "health advocates" OR "health care advocate" OR "health care advocates" OR "healthcare advocate" OR "healthcare advocates" OR "outreach educator" OR "outreach educators" OR "peer health educator" OR "peer health educators" OR "peer health care educator" OR "peer health care educators" OR "peer healthcare educator" OR "peer healthcare educators" OR "nonphysician health worker" OR "nonphysician health workers" OR "nonphysician health care worker" OR "nonphysician health care workers" OR "nonphysician healthcare worker" OR "nonphysician healthcare workers" OR "non-physician health worker" OR "non-physician health workers" OR "non-physician health care worker" OR "non-physician health care workers" OR "non-physician healthcare worker" OR "non-physician healthcare workers" OR NPHW OR NPHWs OR "health auxiliary" OR "health auxiliaries" OR "health care auxiliary" OR "health care auxiliaries" OR "healthcare auxiliary" OR "healthcare auxiliaries" OR "medical auxiliary" OR "medical auxiliaries" OR "medical care auxiliary" OR "medical care auxiliaries" OR "auxiliary health worker" OR "auxiliary health workers" OR "auxiliary health care worker" OR "auxiliary health care workers" OR "auxiliary healthcare worker" OR "auxiliary healthcare workers" OR "social health activist" OR "social health activists" OR "social health care activist" OR "social health care activists" OR "social healthcare activist" OR "social healthcare activists" OR "village health worker" OR "village health workers" OR "village health care worker" OR "village health care workers" OR "village healthcare worker" OR "village healthcare workers"**)**

AND

((mh:SP2.021.167.010.120*) OR videoconferenc* OR "video conference" OR "video conferences" OR "video conferencing" OR "video visit" **OR** "video visits" **OR** "video visitation" OR "video visitations" **OR** "virtual visit" **OR** "virtual visits" **OR** "virtual visitation" OR "virtual visitations" **OR** "virtual intervention" OR "virtual interventions" OR "remote consult" OR "remote consults" OR "remote consultation" OR "remote consultations" OR "remote consulting" OR "video consult" OR "video consults" OR "video consultation" OR "video consultations" OR "video consulting" OR "videoconsult" OR "videoconsults" OR "videoconsultation" OR "videoconsultations" OR "videoconsulting" OR "TIME intervention" OR "TIME interventions" OR (((mh:H02.403.840*) OR telemedicine OR telehealth OR ehealth OR "e-health" OR "remote care") AND (video* OR virtual OR Zoom OR Skype OR FaceTime)) **)**

Global Health; EBSCO

	**Concept: Community Health**	**Concept: Videoconferencing**
Descriptors (DE)	DE "medical auxiliaries"	
Free text terms	CHW<br>CHWs<br>((community W1 (health OR "health care" OR healthcare)) W1 (worker* OR advisor* OR agent* OR aid* OR officer* OR practitioner* OR representative*))<br>(((lay OR nonphysician OR "non-physician" OR auxiliary OR village) W1 (health OR "health care" OR healthcare)) W1 worker*)<br>((health OR 'health care' OR healthcare) W1 (promoter* OR advocate*))<br>promotore*<br>promotora*<br>((outreach OR (peer W1 (health OR "health care" OR healthcare))) W1 educator*)<br>NPHW<br>((health OR 'health care' OR healthcare OR medical OR 'medical care') W1 auxiliar*)<br>(('social health' OR 'social healthcare' OR 'social health care') W1 activist*)	videoconferenc*<br>"video conferenc*"<br>"video visit*"<br>"virtual visit*"<br>"virtual intervention*"<br>"remote consult*"<br>"video consult*"<br>"videoconsult*"<br>"TIME intervention*"<br>(((MH Telehealth) OR telemedicine OR telehealth OR ehealth OR "e-health" OR "remote care") AND (video* OR virtual OR Zoom OR Skype OR FaceTime))

((DE "medical auxiliaries") OR CHW OR CHWs OR ((community W1 (health OR "health care" OR healthcare)) W1 (worker* OR advisor* OR agent* OR aid* OR officer* OR practitioner* OR representative*)) OR (((lay OR nonphysician OR "non-physician" OR auxiliary OR village) W1 (health OR "health care" OR healthcare)) W1 worker*) OR ((health OR 'health care' OR healthcare) W1 (promoter* OR advocate*)) OR promotore* OR promotora* OR ((outreach OR (peer W1 (health OR "health care" OR healthcare))) W1 educator*) OR NPHW OR ((health OR 'health care' OR healthcare OR medical OR 'medical care') W1 auxiliar*) OR (('social health' OR 'social healthcare' OR 'social health care') W1 activist*)**)**

AND

(videoconferenc* OR "video conferenc*" OR "video visit*" **OR** "virtual visit*" OR "virtual intervention*" OR "remote consult*" OR "video consult*" **OR** "videoconsult*" OR "TIME intervention*" OR ((telemedicine OR telehealth OR ehealth OR "e-health" OR "remote care") AND (video* OR virtual OR Zoom OR Skype OR FaceTime)) **)**

Limits: on the Advanced Search screen, make sure that "Apply related words", "Also search within the full text of the articles", and "Apply equivalent subjects" are all un-checked.

Cochrane

	Concept: Community Health	Concept: Videoconferencing
Subject Headings [mh]	[mh "community health workers"]	[mh Videoconferencing]
Free text terms	CHW<br>CHWs<br>((community NEAR/1 (health OR "health care" OR healthcare)) NEAR/1 (worker* OR advisor* OR agent* OR aid* OR officer* OR practitioner* OR representative*))<br>(((lay OR nonphysician OR "non-physician" OR auxiliary OR village) NEAR/1 (health OR "health care" OR healthcare)) NEAR/1 worker*)<br>((health OR "health care" OR healthcare) NEAR/1 (promoter* OR advocate*))<br>promotore*<br>promotora*<br>((outreach OR (peer NEAR/1 (health OR "health care" OR healthcare))) NEAR/1 educator*)<br>NPHW<br>((health OR "health care" OR healthcare OR medical OR "medical care") NEAR/1 auxiliar*)<br>(("social health" OR "social healthcare" OR "social health care") NEAR/1 activist*)	videoconferenc*<br>"video conferenc*"<br>"video visit*"<br>"virtual visit*"<br>"virtual intervention*"<br>"remote consult*"<br>"video consult*"<br>"videoconsult*"<br>"TIME intervention*"<br>(('telehealth'/exp OR telemedicine OR telehealth OR ehealth OR "e-health" OR "remote care") AND (video* OR virtual OR Zoom OR Skype OR FaceTime))

ID	Search	Hits
#1	[mh "community health workers"]	557
#2	CHW OR CHWs OR ((community NEAR/1 (health OR "health care" OR healthcare)) NEAR/1 (worker* OR advisor* OR agent* OR aid* OR officer* OR practitioner* OR representative*)) OR (((lay OR nonphysician OR "non-physician" OR auxiliary OR village) NEAR/1 (health OR "health care" OR healthcare)) NEAR/1 worker*) OR ((health OR "health care" OR healthcare) NEAR/1 (promoter* OR advocate*)) OR promotore* OR promotora* OR ((outreach OR (peer NEAR/1 (health OR "health care" OR healthcare))) NEAR/1 educator*) OR NPHW OR ((health OR "health care" OR healthcare OR medical OR "medical care") NEAR/1 auxiliar*) OR (("social health" OR "social healthcare" OR "social health care") NEAR/1 activist*)	2841
#3	#1 OR #2	2841
#4	[mh Videoconferencing]	245
#5	videoconferenc* OR "video conferenc*" OR "video visit*" OR "virtual visit*" OR "virtual intervention*" OR "remote consult*" OR "video consult*" OR "videoconsult*" OR "TIME intervention*" OR ((telemedicine OR telehealth OR ehealth OR "e-health" OR "remote care") AND (video* OR virtual OR Zoom OR Skype OR FaceTime))	3114
#6	#4 OR #5	3140
#7	#3 AND #6	56

Google search

"community health worker" "visit" "virtual" site:gov

"community health worker" "visit" "virtual" site:org

GODORT IGO/NGO custom Google search
(http://www.google.com/cse/home?cx=012681683249965267634%3Aq4g16p05-ao)

"community health worker" "visit" "virtual"

NIH RePORTER (https://reporter.nih.gov/)

"RePORTER is an electronic tool that allows users to search a repository of both intramural and extramural NIH-funded research projects and access publications and patents resulting from NIH funding."

("community health worker*" OR "community health care worker*" OR "community healthcare worker*" OR CHW OR CHWs OR "community health advisor*" OR "community health care advisor*" OR "community healthcare advisor*" OR "community health agent*" OR "community health care agent*" OR "community healthcare agent*" OR "community health aid*" OR "community health care aid*" OR "community healthcare aid*" OR "community health officer*" OR "community health care officer*" OR "community healthcare officer*" OR "community health practitioner*" OR "community health care practitioner*" OR "community healthcare practitioner*" OR "community health representative*" OR "community health care representative*" OR "community healthcare representative*" OR "lay health worker*" OR "lay health care worker*" OR "lay healthcare worker*" OR "health promoter*" OR "health care promoter*" OR "healthcare promoter*" OR promotore* OR promotora* OR "health advocate*" OR "health care advocate*" OR "healthcare advocate*" OR "outreach educator*" OR "peer health educator*" OR "peer health care educator*" OR "peer healthcare educator*" OR "nonphysician health worker*" OR "nonphysician health care worker*" OR "nonphysician healthcare worker*" OR "non-physician health worker*" OR "non-physician health care worker*" OR "non-physician healthcare worker*" OR NPHW* OR "health auxiliar*" OR "health care auxiliar*" OR "healthcare auxiliar*" OR "medical auxiliar*" OR "medical care auxiliar*" OR "auxiliary health worker*" OR "auxiliary health care worker*" OR "auxiliary healthcare worker*" OR "social health activist*" OR "social health care activist*" OR "social healthcare activist*" OR "village health worker*" OR "village health care worker*" OR "village healthcare worker*"**) AND (**videoconferenc* OR "video conferenc*" OR "video visit*" OR "virtual visit*" OR "virtual intervention*" OR "remote consult*" OR "video consult*" OR "videoconsult*" OR "TIME intervention*" OR ((telemedicine OR telehealth OR ehealth OR "e-health" OR "remote care") AND (video* OR virtual OR Zoom OR Skype OR FaceTime)) **)**

Patient-Centered Outcomes Research Institute (PCORI) - https://www.pcori.org/explore-our-portfolio?keyword=

Studies on comparative clinical effectiveness research focusing on outcomes important to patients

Search on "community health workers" and select Telemedicine checkbox – 4 results

Databases searched with 0 results

Social Work Abstracts (EBSCO)

(CHW OR CHWs OR ((community W1 (health OR "health care" OR healthcare)) W1 (worker* OR advisor* OR agent* OR aid* OR officer* OR practitioner* OR representative*)) OR (((lay OR nonphysician OR "non-physician" OR auxiliary OR village) W1 (health OR "health care" OR healthcare)) W1 worker*) OR ((health OR 'health care' OR healthcare) W1 (promoter* OR advocate*)) OR promotore* OR promotora* OR ((outreach OR (peer W1 (health OR "health care" OR healthcare))) W1 educator*) OR NPHW OR ((health OR 'health care' OR healthcare OR medical OR 'medical care') W1 auxiliar*) OR (('social health' OR 'social healthcare' OR 'social health care') W1 activist*))

AND

(videoconferenc* OR "video conferenc*" OR "video visit*" OR "virtual visit*" OR "virtual intervention*" OR "remote consult*" OR "video consult*" OR "videoconsult*" OR "TIME intervention*" OR (('telehealth'/exp OR telemedicine OR telehealth OR ehealth OR "e-health" OR "remote care") AND (video* OR virtual OR Zoom OR Skype OR FaceTime)))

Social Services Abstracts (ProQuest)

noft(CHW OR CHWs OR ((community PRE/1 (health OR "health care" OR healthcare)) PRE/1 (worker* OR advisor* OR agent* OR aid* OR officer* OR practitioner* OR representative*)) OR (((lay OR nonphysician OR "non-physician" OR auxiliary OR village) PRE/1 (health OR "health care" OR healthcare)) PRE/1 worker*) OR ((health OR "health care" OR healthcare) PRE/1 (promoter* OR advocate*)) OR promotore* OR promotora* OR ((outreach OR (peer PRE/1 (health OR "health care" OR healthcare))) PRE/1 educator*) OR NPHW OR ((health OR "health care" OR healthcare OR medical OR "medical care") PRE/1 auxiliar*) OR (("social health" OR "social healthcare" OR "social health care") PRE/1 activist*))

AND

noft(videoconferenc* OR "video conferenc*" OR "video visit*" OR "virtual visit*" OR "virtual intervention*" OR "remote consult*" OR "video consult*" OR "videoconsult*" OR "TIME intervention*" OR ((telemedicine OR telehealth OR ehealth OR "e-health" OR "remote care") AND (video* OR virtual OR Zoom OR Skype OR FaceTime)))

Sociological Abstracts (ProQuest)

noft(CHW OR CHWs OR ((community PRE/1 (health OR "health care" OR healthcare)) PRE/1 (worker* OR advisor* OR agent* OR aid* OR officer* OR practitioner* OR representative*)) OR (((lay OR nonphysician OR "non-physician" OR auxiliary OR village) PRE/1 (health OR "health care" OR healthcare)) PRE/1 worker*) OR ((health OR "health care" OR healthcare) PRE/1 (promoter* OR advocate*)) OR promotore* OR promotora* OR ((outreach OR (peer PRE/1 (health OR "health care" OR healthcare))) PRE/1 educator*) OR NPHW OR ((health OR "health care" OR healthcare OR medical OR "medical care") PRE/1 auxiliar*) OR (("social health" OR "social healthcare" OR "social health care") PRE/1 activist*))

AND

noft(videoconferenc* OR "video conferenc*" OR "video visit*" OR "virtual visit*" OR "virtual intervention*" OR "remote consult*" OR "video consult*" OR "videoconsult*" OR "TIME intervention*" OR ((telemedicine OR telehealth OR ehealth OR "e-health" OR "remote care") AND (video* OR virtual OR Zoom OR Skype OR FaceTime)))
