# Supplementary material for: Community Health Worker Videoconferencing Interventions for Disease Management and Health Promotion: Protocol for a Scoping Review
Source: JMIR Res Protoc. 2024 Nov 7;13:e55160. doi: 10.2196/55160 (PMC11582480; doi:10.2196/55160)
Supplement: Multimedia Appendix 3 [file resprot_v13i1e55160_app3.pdf]

Data Extraction Elements

This is a Multimedia Appendix to a full manuscript published in the J Med Internet Res. titled "Community health worker videoconferencing interventions for disease management and health promotion: A protocol for a scoping review"

1. Title
2. Last name of first author
3. Year of Publication
4. Journal
5. Meets inclusion criteria? (Y/N)
6. Reason for exclusion (if applicable)
7. Language
8. Country
9. City/State or Region
10. Study design
11. Description of CHW (agency, education/training, qualifications, ethnicity, age, gender, etc)
12. Description of target population (gender, age, race, ethnicity, location, SES, etc)
13. Target disease process or health issue
14. Intervention activities
15. Device loan (Y/N)
16. Intervention medium (use same words as the article)
17. Telehealth location for participant
18. Telehealth location for CHW
19. Results of intervention (if applicable)
20. Barriers to virtual intervention
21. Facilitators of virtual intervention
22. Perceived benefits of virtual intervention
23. Perceived drawbacks of virtual intervention
24. Advice/suggested best practices for virtual intervention
25. Other Notes
